# Evaluating effectiveness and comparative costs of hepatitis C virus self-testing service delivery models in Vietnam: A cross-sectional study

**DOI:** 10.1371/journal.pgph.0005365

**Published:** 2025-11-03

**Authors:** Bao Vu Ngoc, Minh Tran Hung, Huong Phan Thi Thu, Khoa Nguyen Trong, Huong Nguyen Mai, Kim Do Tuan, An Nguyen Le Thanh, Lien Tran Thi Huong, An Tran Khanh, Giang Le Thu, Chau Pham Van, Krista Granger, Karin Hatzold, Yasmin Dunkley, Cheryl Case Johnson, Niklas Luhmann, Kimberly Green

**Affiliations:** 1 PATH, Hanoi, Vietnam; 2 Center for Creative Initiatives in Health and Population, Hanoi, Vietnam; 3 Vietnam Administration of HIV/AIDS Control, Ministry of Health, Hanoi, Vietnam; 4 Vietnam Administration of Medical Services, Ministry of Health, Hanoi, Vietnam; 5 PATH, Washington DC, United States of America; 6 Population Services International, Washington DC, United States of America; 7 London School of Hygiene and Tropical Medicine, London, United Kingdom; 8 World Health Organization, Geneva, Switzerland; McGill University, CANADA

## Abstract

Hepatitis C virus self-testing (HCVST) has been shown to reach people who may not otherwise test. We conducted a cross-sectional survey to assess the effectiveness and costs of different HCVST distribution models among key populations (KPs) and people living with HIV (PLHIV) in Hanoi and Ho Chi Minh City, Vietnam, between September 2023 and April 2024. We engaged eight community-based organizations (CBOs) and 10 public and private clinics in offering HCVST using oral fluid-based HCV rapid antibody tests along with standard or provider-led HCV testing (HCVT). HCVST effectiveness was assessed by the proportion of first-time testers, HCV positivity yield, and linkage to care. Outcomes were stratified by distribution model (community, facility, online, secondary distribution) and compared to standard HCVT. Cost per HCV diagnosis was calculated in US dollars. Among 2,882 participants tested for HCV, 1,834 used HCVST and 1,048 used standard HCVT. HCVST users were more likely to be first-time testers compared to those opting for standard testing (67.6% vs. 59,1%; p < 0.001). The highest proportion of first-time testers was reached through secondary distribution (91.4%) and community distribution (83.8%). HCV positivity through HCVST was significantly lower at CBOs but similar at clinics compared to standard testing (11% vs. 16%; p < 0.01; 16.8% vs. 20.8%; p = 0.094). HCVST at CBOs and clinics was more costly than standard testing ($636 vs. $408 and $605 vs. $218). HCVST was still costlier at CBOs but cheaper at clinics compared to standard testing when kit costs decreased to $2 ($417 vs. $218 and $357 vs. $408). HCVST effectively reached people with HCV and more first-time testers, compared to standard testing among KPs and PLHIV. While current HCVST distribution approaches are costlier than standard testing, modest reductions in commodity costs could make services comparable to help achieve Vietnam’s HCV elimination goals and reach underserved populations.

## Introduction

Hepatitis C virus (HCV) is a major public health challenge with an estimated 244,000 deaths globally in 2022 [1]. There are currently 50 million people with an HCV infection, yet only 36% have been diagnosed and only 20% have received curative treatment [1]. Globally, countries are not on track to achieve the 2030 HCV elimination targets - i.e., to diagnose 90% of people with HCV and treat 80% of those diagnosed [1].

Reaching individuals who are not aware of their HCV status is critical to accelerate attainment of the HCV elimination goals, as HCV testing is the entry point to linking people to diagnosis, treatment, and care. Hepatitis C virus self-testing (HCVST) refers to a process in which an individual collects their own specimen (blood or oral fluid), performs a rapid diagnostic test for the presence of HCV antibodies, and then interprets the result, often in a private setting, either alone or with someone they trust was recommended by the World Health Organization (WHO) in 2021 as an additional approach to HCV testing services [2]. Evidence showed that HCVST increases testing uptake and linkage to care, reaching people who may not have otherwise been tested in existing services, and who may prefer self-care options [2–5]. Results of usability and acceptability studies demonstrated that key populations, such as people who inject drugs (PWID) and men who have sex with men (MSM) and the general population can effectively use HCVST [6–8]. A cost-effectiveness study in four settings (Vietnam, Kenya, China, and Georgia) found that although HCVST was more expensive than facility-based HCV testing, it increased the number of people tested, diagnosed and cured than facility-based HCV testing, and that it could be more cost-effective when introduced in populations with higher prevalence [9].

Vietnam is among the top ten countries that account for nearly two thirds of the global burden of viral hepatitis B and C [1]. HCV poses a significant public health challenge for the country [10,11]. Overall, the prevalence of HCV viraemia – people with active infection in need of treatment - is estimated at 1%. The country estimates one million people living with HCV, with 90% of them are unaware of their HCV status [9,12]. People living with HIV (PLHIV) and PWID have the highest HCV seropositivity rate, ranging from 22.9% to 95.8% [13–18]. It is priority for the country to accelerate access to and uptake of HCV testing, diagnosis and treatment among these high-risk populations.

Low coverage of HCV diagnosis and treatment in Vietnam is attributed to multiple factors, such as centralization of viral load testing and treatment, low awareness of the disease among at-risk populations, out-of-pocket payment, and high cost of diagnosis and treatment, and lack of choice for HCV testing options [6,8,10,11,13]. Despite national guidelines that allow lay providers (individuals who deliver healthcare services and have received some form of training, but do not possess formal professional or paraprofessional certifications or tertiary education degrees) to test clients for hepatitis C and B using rapid tests [6,7,19–21], as standard of care in Vietnam, HCV screening is only available at health facilities requiring out-of-pocket payment [8]. HCV viral load testing is centralized at tertiary hospitals and paid for by social health insurance (SHI). Direct-acting antivirals (DAAs) are covered partially (50%) by SHI, with patients expected to pay for the rest. At total cost between $879 and $963 as of August-2024, 50% of DAAs costs remain too high for many HCV-positive individuals [13,22].

Previous studies [3–8,14,23–25] indicate HCVST is acceptable and feasible in key populations and the general population, and lay people can perform the self-test with minimal support. This reflects Vietnam’s own experience with HIV self-testing (HIVST) [26–28]. However, the remaining challenge lies in effectively implementing, positioning and scaling HCVST to complement other HCV testing services and addressing gaps in current coverage. The first critical step towards HCVST introduction and scale-up is to determine where and how to implement HCVST to support scale-up of this intervention.

With this study, we investigate the effectiveness and costs of HCVST service delivery models compared to standard HCV testing using point-of-care (POC) rapid diagnostic test delivered by clinics and CBOs.

## Materials and methods

### Ethics statement

We obtained ethical approval from the WHO Ethics Review Committee (reference number ERC 0003830 dated 15 September 2022), the local Institutional Review Board at Center for Creative Initiatives in Health and Population – CCIHP (reference number 011223/HDPB-CCIHP dated 30 November 2022) and the National Research Ethics Committee (reference number 235/CN-HDDD dated 22 December 2022). All study participants provided written informed consent except for consent waivers for the recipients of HCVST through the secondary distribution delivery model.

### Study design

We conducted a cross-sectional study from September 2023 to April 2024 to assess the effectiveness and costs of HCVST distribution models compared to standard POC rapid HCV testing delivered by clinics and CBOs. A total of 20 study sites participated in this study. They included: 1) eight CBOs offering community-based and secondary distribution HCVST models along with standard community-based HCV rapid testing; 2) ten primary health care (PHC) public and private clinics (i.e., 3 methadone maintenance treatment-MMT, 3 antiretroviral thrapy-ART, and 4 pre-exposure prophylaxis-PrEP clinics) providing facility-based HCVST distribution along with standard POC HCV rapid testing; and 3) two KP-led clinics providing online HCVST integrated into online platform (http://xomcauvong.com).

### Description of the intervention

As an integrated approach, we implemented HCVST distribution in all existing settings of HIV and harm reduction services. This included four HCVST service delivery models: (1) Facility-based HCVST distribution (FBD) was integrated into ART outpatient clinics, MMT facilities, and private KP-led PrEP clinics; (2) Community-based HCVST distribution (CBD) was provided by community-based organizations (CBOs); (3) Secondary distribution of HCVST (SD) was offered by CBOs to sexual partners and/or injecting peers of clients who received HCVST at CBOs (social network approach); and (4) Online distribution of HCVST was administered by private KP-led clinic using an existing online platform (http://xomcauvong.com) to reach and administer home delivery of HCVST kits to clients who frequently visit online platforms, such as young people under 25 years of age. The clinics offered a choice of facility-based HCVST distribution or standard POC rapid HCV testing delivered by clinics (clinic HCVST) for clients seeking or using MMT, ART and/or PrEP services, while the CBOs offered a choice of community-based HCVST distribution or HCV rapid testing provided by CBOs (CBO HCVST) for clients receiving condoms, needles & syringes and/or community HIV testing. Secondary distribution and online distribution approaches have been implemented for HIVST but not yet for HCVST in Vietnam. In this study secondary distribution of HCVST was implemented through engagement of primary HCVST clients at CBOs. Each primary HCVST client distributed up to three kits to their sexual and/or injecting partners who they thought could benefit from HCVST. CBO service providers followed up clients who received the HCVST kits via telephone for up to four weeks to check if they performed the self-test and/or visited the designated clinics for HCV confirmatory testing and/or treatment initiation. Online distribution was administered by two KP-led clinics, leveraging the existing HIVST online platform (http://xomcauvong.com). Clients could access online sites via a personal computer, smartphone, or tablet, complete a short questionnaire for risk assessment and then place an online order for home delivery of HCVST kits through standard post mail, or Grab/Be/Gojek services (mobile app-based delivery/courier companies), or self-pickup at a clinic within two weeks. The HCVST kit consisted of the test, instructions for use, referral slip, and information about other supporting materials, such as access to live chat and a call center for answering questions about testing. Clients were supported to link to care through accompanied referrals if requested either at the point of testing reactive if self-testing on-site, or within four weeks if self-testing off-site. The only deviation from this was through the clinic’s provision where passive referrals were used with clients with reactive test results on-site referred to confirmatory testing and treatment using a standard referral slip.

### HCVST kits

We used the WHO prequalified OraQuick HCV rapid antibody test (OraSure Technologies, PA, USA) [16,18,29–33] alongside manufacturer adapted modified Instructions for Use (IFU).

### Study eligibility

Any PLHIV, KPs (i.e., PWID, MSM, female sex workers -FSW, transgender women-TGW) and their sexual and/or injecting partners seeking HIV or harm reduction services at the study sites, 18 years or older with unknown HCV status or not tested for HCV in the past six months, able to provide written consent for participation in the study, with access to a reliable phone for follow-up were eligible for the study,

### Sample size and sampling

For the cross-sectional study, a sample size of up to 524 participants was required for each HCV testing model (facility-based, community-based, or secondary distribution HCVST, clinic or CBO PL-HCVT) to measure the effectiveness of the intervention in reaching first-time HCV testers. This sample size enabled a precision estimate of first-time testers at 30% to 90% accuracy [34]. For the online HCVST model, we estimated a sample size of 262 participants based on the assumption that first-time HCV testers accounted for 50% of total online HCVST clients, to 90% accuracy. A total sample of 2,882 participants was required for all HCV testing models.

All clients seeking community-based, facility-based, and online HIV or harm reduction services during the study period were invited to participate in this study. The “take all” method was applied to recruit study participants. The recruitment was consecutively carried out until it reached the expected sample size for each testing model.

### Data collection

Study participants were recruited for this study in the period from 04 September 2023–28 February 2024. Quantitative data was collected through: (1) A cross-sectional survey questionnaire administered with study participants. Structured interviews on-site or via telephone for those who opted for HCVST secondary distribution and online distribution; (2) An e-logbook maintained client records when using HCV services; (3) An online HCVST web-based application captured self-reported information (as the first part of the questionnaire above) on people accessing the website and opting for HCVST; and (4) An Excel worksheet recorded expenditures and costs related to HCV testing program implementation to serve as a basis for the cost analysis. We obtained data for the costing analysis through review of administrative records of 18 facilities (8 CBOs and 10 clinics) involved in implementing four HCVST service delivery models over eight months.

### Analysis approach

The primary outcome of interest was the effectiveness of the different HCVST delivery models. Based on evidence from previous cost-effective studies on HIVST and HCVST [9,35], we measured effectiveness of an HCV self-testing model by the proportion of first-time testers, positivity yield and linkage to care compared to standard/provider-led HCV testing models. In this study, first-time testers refer to individuals who have never been tested for HCV before using any method, including self-testing or testing at a clinic or other healthcare setting. This target group is particularly important among populations that are at risk (e.g., PWID, MSM and PLHIV) but never tested due to being unaware of risk, fear of stigma and discrimination, or inaccessibility of existing HCV testing services. The cost of the HCVST service delivery model was measured by cost per HCV test and cost per diagnosis.

Data was analyzed using descriptive statistics and multivariable logistic regression models in SPSS Version 22.0 (based on license availability) and reported in accordance with STROBE (Strengthening the Reporting of Observational Studies in Epidemiology) and CHEERS (Consolidated Health Economic Evaluation Reporting Standards 2022) guidelines. We summarised participant characteristics as proportions, and medians (interquartile range [IQR]), as appropriate, and compared demographic variables between those that opted for self-testing and those that tested in clinics or CBOs using chi square tests of association and Mann-Whitney U tests for categorical data and medians. To assess effectiveness of service delivery models in reaching first-time testers (primary outcome), we compared the overall proportion of first-time testers between HCVST models and standard HCV testing models. We then calculated the proportion of first-time testers by key population group.

We explored whether HCVST was effective in reaching first-time testers, and if so, what were the most effective HCVST service delivery models when compared to standard HCV testing. To investigate whether the testing delivery models (standard testing vs HCVST) were independently associated with first-time testing, we conducted multivariable logistic regression analysis. We adjusted our final model by age group (18–29, 30–49, and 50+), marital status (unmarried, married, living with a partner, and divorced/separated/widowed), key population identity (MMT PWID and non-MMT PWID, MSM, PLHIV, FSW, sex partners), occupation (unemployed, employed, student, self-employed/freelancer), education (primary school, secondary school, high school, and under graduated) and income (under 5 million, 5–10 million, 11–15 million, and 15 + million VND). Variables were grouped in line with national programme requirements and stability and selected for the final model inclusion because of a priori hypothetical confounding, with significance assessed using Wald and likelihood ratio tests.

To assess effectiveness of delivery models in linking people to care, we compared the care cascades of people testing HCV-reactive, confirmed HCV positive, and linked to DAAs by HCVST delivery models and standard/provider-led HCV testing models. We then calculated the proportion HCV-reactive by key population group.

### Costing analysis

Finally, we estimated the costs of providing different HCV testing models, including facility-based distribution, community-based distribution (primary distribution) and secondary distribution, online distribution of HCVST kits, and SOC clinic HCVT or CBO HCVT. As we aimed to compare costs of different HCV test delivery models, we adopted a healthcare provider perspective in our costing, which estimated the incremental costs to the facility (clinic or CBO) for providing HCV tests using HCVT or HCVST kits. We used a bottom-up micro-costing approach [36] for the estimation in which we itemized all incremental expenses for the facility in all cost categories, including capital costs (i.e., infrastructure and equipment if additional purchase was needed) and recurrent costs (i.e., test kits, and supplies for testing, overhead, transportation, personnel, training and communications). Costs of confirmation HCV and DAA treatment were not included as they are assumed standards for all patients across different HCV testing models. Vietnam currently applies a two-step HCV confirmation approach that involves an initial screening with an HCV antibody test, followed by an HCV RNA test (NAT) for confirmation if the antibody test is reactive. S1 Text described the cost categories that were examined in this analysis. For the online distribution model, there is a capital cost to develop the online platform. This capital cost is added to the monthly total cost by estimating the monthly cost from annualized capital cost using the common discount rate of 3% and assumed lifespan of 10 years following expert’s opinion. Cost data were in VND and converted to 2023 US dollars using the exchange rate of 1 USD = 24,000 VND as used by the finance department throughout the project.

The costs of all categories were added together to create a total cost for each testing model. We estimated the cost per test by dividing the total cost with the total number of tests performed in each testing model. Similarly, the cost per diagnosis was calculated by dividing the total cost with the total number of HCV diagnosis with confirmed tests in each testing model. We calculated the incremental costs per month for the facility by adding the average monthly amount of all cost categories. Because the unit costs of the test kits were substantially different between provider-led testing and self-testing costs, we performed sensitivity analysis in which we varied unit costs of HCVST to $1, $2 and $5 to see how the test-kit cost affected cost per diagnosis.

## Results

### Demographic characteristics of study participants

Data was collected on 2,882 clients in Hanoi and HCMC, including 1,834 who used HCVST and 1,048 using standard clinic and CBO HCV testing. Clients opting for HCVST were significantly younger than those opting for standard testing (median age: 36 and 39, respectively; p-value <0.001). There was no difference in gender between clients opting for HCVST compared to standard testing. Clients opting for HCVST had a higher likelihood of being unmarried, student, MSM and sex partners, higher education than standard HCV testing clients, while clients opting for standard testing had higher proportion of being married, self-employed or freelancer, PWID on MMT (MMT PWID) and PLHIV on ART (ART PLHIV) than HCVST clients (Table 1).

**Table 1:** Demographic characteristics of study participants.

Characteristic	HCVST	Standard HCVT	Total	p-value
	(n = 1,834)	(n = 1,048)	(n = 2,882)	
Age in years, median (IQR)	36 (27-43)	39 (30-47)	37 (28-44)	0.000^a***^
18-29	588 (32.1%)	254 (24.2%)	842 (29.2%)	0.000^b***^
30-39	507 (27.7%)	280 (26.8%)	787 (27.3%)	
40-49	506 (27.6%)	378 (36.1%)	884 (30.7%)	
50+	233 (12.6%)	136 (12.9%)	369 (12.8%)	
Gender§				
Male	1002 (76.5%)	790 (75.4%)	1792 (76.0%)	0.499
Female	301 (23.0%)	255 (24.3%)	556 (23.6%)	
Transgender	7 (0.5%)	3 (0.3%)	10 (0.4%)	
Marital status				
Unmarried	847 (46.2%)	394 (37.6%)	1241 (43.1%)	0.000^b***^
Married	693 (37.8%)	469 (44.8%)	1162 (40.3%)	
Living with a partner	151 (8.2%)	83 (7.9%)	234 (8.1%)	
Divorced, separated or widowed	143 (7.8%)	102 (9.7%)	245 (8.5%)	
Education				
Primary	160 (8.7%)	100 (9.5%)	260 (9.0%)	0.000^b***^
Secondary	546 (29.8%)	336 (32.1%)	882 (30.6%)	
High school	540 (29.4%)	385 (36.7%)	925 (32.1%)	
University and higher	588 (32.1%)	227 (21.7%)	815 (28.3%)	
Occupations				
Employed	533 (29.1%)	274 (26.1%)	807 (28%)	0.000^b***^
Student	186 (10.1%)	49 (4.7%)	235 (8.2%)	
Self-employed/Freelancer	837 (45.6%)	582 (55.5%)	1419 (49.2%)	
Unemployed	109 (5.9%)	62 (5.9%)	171 (5.9%)	
Others	169 (9.2%)	81 (7.7%)	250 (8.7%)	
Monthly income				
Under 5 million (208 USD)	640 (34.9%)	347 (33.1%)	987 (34.2%)	0.002^b**^
5–10 million (208–417 USD)	852 (46.5%)	509 (48.6%)	1361 (47.2%)	
Above 10–15 million (458–625 USD)	217 (11.8%)	138 (13.2%)	355 (12.3%)	
Above 15 million (625 USD)	94 (5.1%)	53 (5.1%)	147 (5.1%)	
No answer	31 (1.7%)	1 (0.1%)	32 (1.1%)	
Population group				
Non-MMT PWID	497 (27.1%)	285 (27.2%)	782 (27.1%)	0.000^b***^
MMT PWID	196 (10.7%)	187 (17.8%)	383 (13.3%)	
MSM	387 (21.1%)	165 (15.7%)	552 (19.2%)	
ART PLHIV	314 (17.1%)	235 (22.4%)	549 (19.0%)	
FSW	105 (5.7%)	80 (7.6%)	185 (6.4%)	
Sex partner	216 (11.8%)	83 (7.9%)	299 (10.4%)	
Others	119 (6.5%)	13 (1.2%)	132 (4.6%)	

Note: §Secondary distribution sample (n = 524) excluded; ^a^ Mann-Whitney U test; ^b^ Chi square test; **p < 0.01; ***p < 0.001.

### First-time HCV testers

Overall, HCVST models reached a higher proportion of first-time HCV testers than standard HCV testing (67.6% vs. 59.1%, respectively; p < 0.001) in which HCVST secondary distribution and community-based distribution had a higher proportion of first-time testers than online distribution and facility-based distribution (91.4% and 83.8% vs. 48.9% and 36.8%, respectively) (Table 2).

**Table 2:** Proportion of first-time HCV testers by testing model.

Testing model	HCVST				Standard HCV testing		p value
	Community-based distribution	Facility-based distribution	Secondary distribution	Online distribution	CBO	Clinic	
	n = 524	n = 524	n = 524	n = 262	n = 525	n = 523	
First-time tester (Yes)	83.8%	36.8%	91.4%	48.9%	81.1%	36.9%	0.000***
Ever tested (No)	16.2%	63.2%	8.6%	51.1%	18.9%	63.1%	

Chi-square: ***p < 0.001.

**Fig 1:**
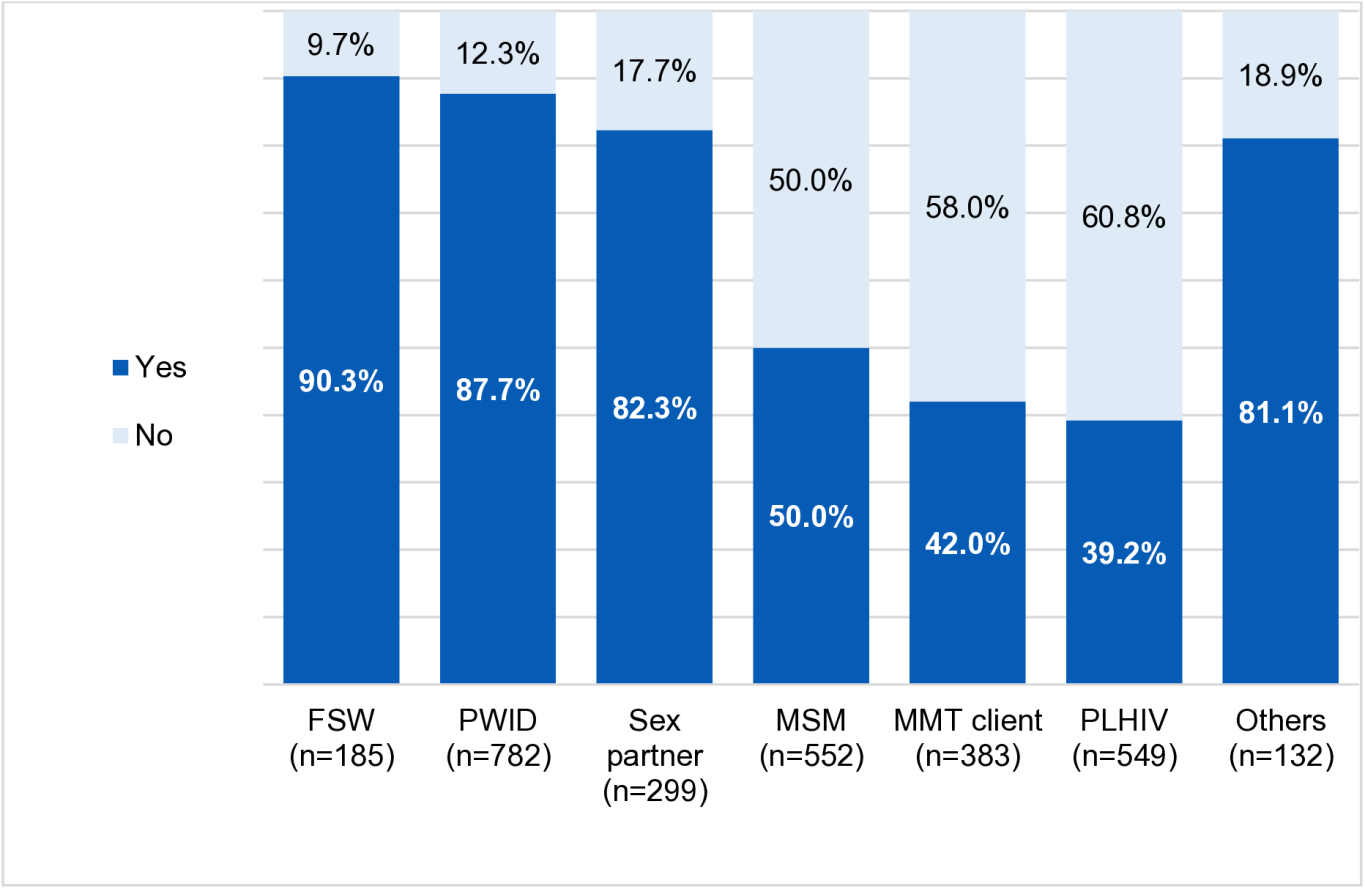
Proportion of first-time testers by key population group.

By population group, the proportion of first-time testers was higher among FSW, PWID and their sexual partners than MSM, PLHIV and MMT users (90.3%, 87.7% and 82.3% vs. 50%, 42% and 39.2%, respectively; p < 0.001) (Fig 1).

### Factors associated with first-time testing

Univariate analysis results revealed that first-time testers were more common among PWID, FSW and sex partner clients (Table 3). A multivariable logistic regression analysis adjusted for age group, marital status, education level, occupation, income, population group, and testing model confirmed a significant association between first-time HCV testing and being a PWID (adjusted odds ratio [aOR]: 7.15; 95% confidence interval [CI]: 5.45-9.37), FSW (aOR: 9.28; 95% CI: 5.55-15.51) or a sex partner thereof (aOR: 4.64; 95% CI: 3.3-6.52), as well as amongst those testing within community-based distribution (aOR: 8.83; 95% CI: 6.59-11.83) secondary distribution models (aOR: 18.2; 95% CI: 12.78-25.92) or CBO HCVT (aOR: 7.36; 95% CI: 5.55-9.75). In contrast, PWID on MMT and PLHIV on ART, who often received HCV testing as standard of care, were less likely to be first-time HCV testers in this study. Similarly, those who were aged 30 years and older, who had higher education or monthly income less than 15 million VND ($625) were also less likely to be first-time testers.

**Table 3:** Factors associated with first-time HCV testing.

Factors	Univariate regression		Multivariate regression	
	Odds ratio (95% CI)	p value	Adjusted odds ratio (95% CI)	p value
Age group				
18-29	Reference			
30-49	1.11 (0.93-1.32)	0.245	0.67 (0.51-0.88)	0.005**
50+	0.94 (0.73-1.21)	0.616	0.38 (0.26-0.57)	0.000***
Marital status				
Unmarried	Reference			
Married	1.72 (1.45-2.03)	0.000	1.1 (0.86-1.41)	0.448
Living with a partner	1.73 (1.28-2.35)	0.000	0.79 (0.54-1.15)	0.22
Divorced, separated, widowed	1.65 (1.23-2.21)	0.001	0.84 (0.57-1.24)	0.37
Occupation				
Employed	Reference			
Student	1.15 (0.85-1.56)	0.353	1.36 (0.88-2.09)	0.166
Self-employed/ Freelancer	1.3 (1.08-1.55)	0.005	1.01 (0.79-1.29)	0.946
Unemployed	1.27 (0.9-1.8)	0.172	0.74 (0.45-1.19)	0.214
Others	1.25 (0.93-1.68)	0.145	0.98 (0.67-1.44)	0.922
Education				
Primary school	Reference			
Secondary school	0.94 (0.70-1.27)	0.689	0.73 (0.5-1.05)	0.091
High school	1.22 (0.9-1.65)	0.195	1.13 (0.77-1.66)	0.543
Under Graduated	0.43 (0.32-0.58)	0.000	0.52 (0.33-0.81)	0.004**
Monthly income in VND				
Under 5 million ($208)	Reference			
5–10 million ($208–416)	0.77 (0.65-0.92)	0.004	0.66 (0.51-0.85)	0.001**
11–15 million ($458–625)	0.44 (0.34-0.56)	0.000	0.58 (0.4-0.84)	0.004**
Above 15 million ($625)	0.49 (0.35-0.7)	0.000	1.02 (0.64-1.64)	0.924
Not answer	1.27 (0.56-2.85)	0.568	1.99 (0.81-4.89)	0.132
Key Population group				
MSM	Reference			
MMT PWID	0.73 (0.56-0.94)	0.017	0.66 (0.44-0.98)	0.038*
Non-MMT PWID	7.15 (5.45-9.37)	0.000***	1.98 (1.31-2.97)	0.001**
ART PLHIV	0.64 (0.51-0.82)	0.000	0.44 (0.32-0.62)	0.000***
FSW	9.28 (5.55-15.51)	0.000***	2.37 (1.31-4.27)	0.004**
Sex partner	4.64 (3.3-6.52)	0.000***	1.69 (1.11-2.57)	0.015*
Others	4.28 (2.69-6.82)	0.000	2.18 (1.3-3.68)	0.003
Testing model				
Clinic HCVT	Reference			
CBO HCVT	7.36 (5.55-9.75)	0.000***	3.97 (2.82-5.57)	0.000***
Secondary distribution	18.2 (12.78-25.92)	0.000***	10.19 (6.82-15.21)	0.000***
Online distribution	1.63 (1.21-2.21)	0.001	0.96 (0.67-1.4)	0.849
Facility-based distribution	1.00 (0.78-1.28)	0.981	0.99 (0.76-1.3)	0.964
Community-based distribution	8.83 (6.59-11.83)	0.000***	5.44 (3.86-7.68)	0.000***

Multivariable logistic regression test: *p < 0.05; **p < 0.01; ***p < 0.001.

### HCV cascade of care

Overall, community-based and facility-based distribution of HCVST yielded as higher rate of HCV positivity as CBO and clinic HCVT (18.1% and 16.8% vs. 16% and 20.8%, respectively), while secondary distribution and online distribution models yielded lower positivity rate (3.8% and 1.5%, respectively) (Table 4). Similarly, the success rate of linkage to care from community-based and facility-based distribution of HCVST was similar to CBO HCVT and clinic HCVT (90.5% and 98.9% vs. 85.7% and 99.1%, respectively). Overall, there was a high rate of people diagnosed with DAA treatment across all HCVST distribution models (range: 96.4-100%). However, secondary distribution and community-based HCVST had a bit lower HCV confirmatory linkage than facility-based and online distribution (80% and 90.5% vs. 98.9% and 100%, respectively).

**Table 4:** Cascade of care by HCV testing model.

HCV testing model	Tested for anti-HCV	Anti-HCV+	Confirmatory testing	Confirmed HCV	Treatment initiation
Community-based distribution	524	95 (18.1%)	86 (90.5%)	55 (64.0%)	53 (96.4%)
Facility-based distribution	524	88 (16.8%)	87 (98.9%)	65 (74.7%)	65 (100.0%)
Secondary distribution	524	20 (3.8%)	16 (80.0%)	11 (68.8%)	11 (100.0%)
Online distribution	262	4 (1.5%)	4 (100.0%)	3 (75.0%)	3 (100.0%)
CBO HCVT	525	84 (16.0%)	72 (85.7%)	44 (61.1%)	42 (95.5%)
Clinic HCVT	523	109 (20.8%)	108 (99.1%)	68 (63.0%)	63 (92.6%)

**Fig 2:**
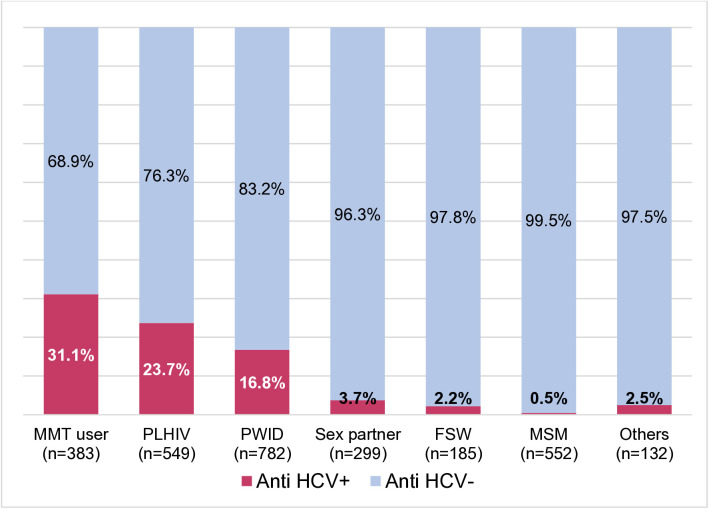
HCV positivity rate by key population group opting for HCVST and standard HCVT.

Among clients opting for HCVST and standard HCVT, HCV positivity rate was significantly higher in MMT users, followed by PLHIV and PWID than in sex partners, FSW, and MSM (31.1%, 23.7%, and 16.8% vs. 3.7%, 2.2%, and 0.5%, respectively; p < 0.001) (Fig 2).

### Cost analysis

Overall, the cost per test through HCVST was higher than standard HCV testing ($15.2 - $19.7 vs. $9.9-$14,5, respectively) (Table 5).

**Table 5:** Comparative cost analysis of HCVST and PL-HCVT models (in US$).

Testing model	Clinics (n = 10)		CBOs (n = 8)			
	Facility-based distribution	Clinic HCVT	Community-based distribution	CBO HCVT	Secondary distribution	Online distribution (n = 2)
**Base case analysis**						
Average cost per test	$17.0	$9.9	$19.6	$14.5	$15.2	$19.7
Proportion of test kit cost in total cost	54.9%	19.2%	43.1%	13.7%	58.9%	40.4%
Average cost per HCV diagnosis	$636	$408	$605	$218	$985	$3,639
Average cost per HCV diagnosis exc. test kit cost	$263	$335	$354	$198	$538	$2,143
HCV positivity rate	8.2%	18.0%	14.8%	14.7%	3.5%	1.0%
Incremental cost per month for the facility	$838	$512	$656	$277	$343	$990
Incremental cost per month for the facility exc. test kit cost	$373	$437	$404	$244	$171	$591

Note: Exchange rate: 1US$ = 24,000 VND; Cost per test kit in base case: US$1.3 for Bioline HCV test and US$7.9 for OraQuick HCV test.

In the base case analysis, the HCVST test kit cost is the main cost driver as it accounted for 40.4% to 58.9% of the total cost in HCVST models compared to from 13.7% to 19.2% in standard HCV testing models. Among HCVST models, the cost per test was lowest in secondary distribution models, followed by facility-based, community-based, and online distribution ($15.2; $17; $19.6 and $19.7). The cost per HCV diagnosis through HCVST was higher than standard HCV testing ($605.4 - $3,639 vs. $217.8 - $408, respectively). Among HCVST model, the cost per HCV diagnosis was lowest in community-based HCVST, followed by facility-based HCVST, secondary distribution and online distribution ($605.4; $635.9; $984.5; and $3,639, respectively).

In the sensitivity analysis, when the HCVST test kit unit cost is reduced to $1, the average cost per test of HCVST is comparable with HCVT in the clinics ($10.1 vs $9.9) and HCVST cost per test is lower than HCVT in CBO settings ($8.3-$12.6 as compared to $14.5). If the HCVST test kit unit cost is less than $5, the cost per HCV diagnosis using HCVST is less than that of using the conventional HCVT in clinic settings ($310-$357 as compared to $408). In CBO settings, the cost per HCV diagnosis using HCVST is greater than that of using HCVT regardless of the HCVST unit cost. Results of the sensitivity analysis are included in S1 Table.

Notably, incremental costs per month for adding HCVST to the clinic were lower than standard clinic HCV testing when excluding the test kit cost compared to including the test kit cost ($373.1 vs. $436.6 compared to $837.8 vs. $512.2, respectively). In contrast, incremental costs per month for adding HCVST to the CBO were unchanged for either exclusion or inclusion of the test kit cost ($404 vs. $243.8 compared to $655.5 vs. $277, respectively). However, incremental costs per month for adding secondary distribution of HCVST to the CBO were lower than the standard CBO testing when excluding the test kit cost compared to including it ($171.3 vs. $243.8 compared to $343.2 vs. $277, respectively).

Table 6 presents the components of the incremental cost per month for facilities to provide HCVST and standard HCV testing. The test-related costs included those for the test kit, supplies for fingerstick blood drawing for standard HCV testing, and for referral form printouts if the facility did not provide HCV confirmatory testing. With the high unit cost of the HSVST kit, the test-related costs were the main cost driver across HCVST delivery models. Costs of personnel were also a major cost driver in HCVST and standard HCV testing models. The staff at the clinics perceived that it took a bit longer time performing standard HCV testing than HCVST, while at CBOs, the staff spent more time on HCVST than HCVT for self-test introduction and user guidance. The online HCVST distribution model had some costs to organize communication events to introduce the new platform and overhead costs to operate the web-based platform.

**Table 6:** Incremental costs per month for different HCV testing models.

Testing model	Clinics (n = 10)		CBOs (n = 8)			
	Facility-based distribution	Clinic HCVT	Community-based distribution	CBO HCVT	Secondary distribution	Online distribution (n = 2)
Incremental cost per month for the facility	$838	$512	$656	$277	$343	$990
Component costs						
Test-related costs	$467	$97	$252	$44	$172	$400
Overhead costs	$10	$17	$59	$56	$3	$88
Transportation costs	$0	$0.03	$7	$1	$1	$4
Personnel costs	$304	$347	$279	$153	$126	$396
Costs for training and communications	$57	$51	$58	$22	$41	$101

Note: Exchange rate: 1US$ = 24,000 VND; Cost per test kit in base case: US$1.3 for Bioline HCV test and US$7.9 for OraQuick HCV test.

## Discussion

This study evaluated the effectiveness of four HCVST service delivery models and compared the effectiveness of community-based and facility-based HCVST with standard HCV testing at CBOs and clinics among key populations and PLHIV in Hanoi and Ho Chi Minh City, Vietnam. The study also conducted comparative cost analysis of HCVST compared to standard HCVT models. Our results indicate that multiple HCVST distribution models were effective in diagnosing people with HCV and reaching first-time testers. In reaching first-time testers, integration of HCVST leads to additional people diagnosed and treated, however this is at a higher cost per diagnosis than standard HCV testing. This finding is consistent with a cost-effectiveness study in four countries including Vietnam that found HCVST increases the number of people tested, diagnosed and treated but at a higher cost – largely driven by the high cost of the test-kit [9].

Our study found that community-based distribution and secondary distribution of HCVST were the most effective at reaching first-time HCV testers, compared to facility-based distribution and online HCVST distribution. However, every model reached novel participants; existing facility-based HCV testing currently reaches and tests PWID on MMT, PLHIV on ART, and MSM on PrEP who frequently visit clinics and are offered HCV testing as standard of care, while community-based distribution and secondary distribution as well as CBO HCVST are well positioned to reach underserved key populations, i.e., PWID not accessing MMT, FSW and sex partners who may not have been reached and tested for HCV in either HIV or HCV services.

We observed that HCV positivity yield was different through HCVST distribution models. The main explanation for this is the ability to reach at-risk populations. For example, facility-based HCVST reached most at-risk populations, i.e., MMT and PLHIV and community-based HCVST reached PWID, while secondary distribution reached sexual/injecting partners and online distribution more likely reached MSM. This suggests that HCVST is an alternative testing option at facilities and a supplement to community-based HCV testing to reach more people who would not have otherwise been tested or diagnosed by existing services. In reaching first-time testers, our findings are consistent with a study in China that reported first-time testers accounted for over half of HCV self-testers among MSM [4]. This suggests that HCVST is an effective strategy to reach first-time HCV testers who have not been in the health system and/or prefer self-care/self-testing options.

Similar to HIV self-testing reported in a systematic review and meta-analysis [37], we found high rates of confirmatory testing and treatment initiation of DAAs among those with reactive HCVST results. Linkage to care was high across distribution models. This success is likely due to the co-location of HCV services at health facilities providing MMT, ART and/or PrEP, and CBO engagement in service delivery. CBOs implemented community-based distribution and secondary distribution models and offered active referrals, such as accompanying clients to or scheduling appointments with health facilities for HCV confirmatory testing and/or treatment registration.

While HCVST offers the potential to expand access to HCV testing, implementing a novel testing strategy incurs additional costs and requires individuals with reactive self-tests to follow up with confirmatory testing and treatment. Previous studies on HIVST revealed that HIVST is cost-effective, and its major drivers include underlying HIV prevalence, testing cost and linkage to care [35]. Similarly, our study found that the cost per HCV diagnosis from HCVST is higher than the standard HCV testing delivered by health facilities or CBOs, and the discrepancy across testing models is mainly driven by HCV positivity rate. It means that the higher the HCV positivity yield, the lower the cost per diagnosis incurred. Among four HCVST service delivery models, community-based and facility-based distribution are considered the least costly compared to secondary distribution and online distribution, and this discrepancy is driven by HCV positivity rate (14.8%, 8.2%, 3.5% and 1%, respectively). This finding is in line with previous study indicating that HCVST is more cost-effective in populations with high prevalence [9]. Our study highlighted HIVST was more cost-effective where it potentially reached most-at risk populations, such as PWID and PLHIV. The cost per HCV diagnosis through HCVST remained higher than standard HCV testing. However, when test kit costs were reduced to $1 or $2, the cost per HCV diagnosis in HCVST was cheaper than standard HCV testing at clinics, but still costlier than standard HCV testing at CBOs. This can be explained by clinic/CBO personnel costs for time devoted for HCVST versus standard HCV testing. At clinics service providers spend less time providing HCVST than standard HCV testing, while at CBOs staff usually spend more time distributing HCVST than offering standard HCV testing to clients. For example, they took additional time to introduce HCVST and instructions for use before giving clients a self-test kit. Efforts to advocate for lower price of HCVST kits are critical to facilitate scale-up of HCVST in Vietnam. Learnings from successful HIV self-testing program in Vietnam are applicable to HCVST. As part of the advocacy efforts, we disseminated research findings to key stakeholders and prepared the paper for publishing in a peer-reviewed journal. Other ongoing efforts include updating the national hepatitis testing guidelines for the inclusion of HCVST; facilitating market authorization of HCVST products in the country; integrating HCVST into hepatitis program plans; and implementing, monitoring and evaluation of HCVST program. This pathway aims to shape HCVST market that enables us to achieve lower prices of HCVST kits in the country.

This study is the first to identify optimal HCVST distribution models in a real-world setting among key populations and PLVIH in Vietnam. Results of the study suggest that selection of optimal HCVST service delivery models should take into consideration the effectiveness of the model and the cost in addition to other factors, such as the preference and the feasibility to sustain and scale.

### Limitations

This study has several limitations. Only oral fluid-based HCVST kits were available to offer in this study. This limited in offering choice for people who opted for HCVST. Convenience and “take all” sampling methods may create a selection bias in recruiting a sample of study participants that may not be representative of the entire key populations and PLHIV. This study did not enable us to collect data on sustained virus response at 12 weeks after treatment (SVR12) to evaluate cure rate of HCV treatment. Due to study time constraints, we did not investigate the costs as part of patient and societal perspectives to depict the overall economic burden and thus, several costs were omitted such as DAA treatment costs, non-medical costs for patients and households such as transportation, or indirect costs such as productivity loss for healthcare utilization. The costing analysis is limited to comparison of costs of healthcare providers providing HCV test services only.

## Conclusions

Findings of the study confirm that HCVST is an effective approach to diagnose people with HCV and reach first-time testers. While all models evaluated reached novel populations and achieved important effects, community-based and secondary distribution of HCVST identified the most people with HCV and the most first-time testers; they also appeared to be more affordable and may incur lower incremental costs when added to existing services in Viet Nam. While the current approaches for distributing HCVST were more costly than standard HCV testing options, modest reductions in commodity costs would make services comparable in price. To achieve HCV elimination goals and reach those underserved by existing health services it is critical to invest in HCVST and prioritize ways to accelerate reductions in commodity costs.

## Supporting information

S1 Text Cost categories and itemized costs included in the analysis. (DOCX)

S1 Table Comparative costs of HCVST and PL-HCVT models in sensitivity analysis (in US$). (XLSX)
