# Supplementary material for: Evaluating effectiveness and comparative costs of hepatitis C virus self-testing service delivery models in Vietnam: A cross-sectional study
Source: PLOS Glob Public Health. 2025 Nov 3;5(11):e0005365. doi: 10.1371/journal.pgph.0005365 (PMC12582494; doi:10.1371/journal.pgph.0005365)
Supplement: S1 Text — (DOCX) [file pgph.0005365.s001.docx]

**S1 Text. Cost categories and itemized costs included in the analysis.**

| **Cost categories** | **Description** |
| --- | --- |
| Capital costs | - Costs for infrastructure and equipment if additional purchase is needed, e.g., costs to develop a platform for online distribution of HCVST. |
| Recurrent costs | - Costs for items used on regular basis |
| Test-related costs | - Test kits (OraQuick HCV rapid antibody test for direct and indirect HCVST; Bioline HCV antibody test for PL-HCVT) - Supplies for testing, including masks, gloves, alcohol, needles, tubes, cotton balls, and bandages. - Printouts referral for confirmation test. |
| Overhead costs | - Additional costs if needed for office and sanitation supplies, drinking water, disposable cups, utilities, waste management, telephone, internet, and server rental. |
| Transportation costs | - Fuel costs or taxi costs for staff if they go with clients for confirmation test. - For CBOs, fuel costs or taxi costs if they go to find cases. - For online distribution, shipping costs of test kits to clients. |
| Personnel costs | - Costs for each position are estimated by salary and time spent on test-related activities including consultation, instruction, testing, result informing, referring to confirmation test, management, training, and communications. |
| Costs for training and communications | - Costs to conduct training and social mobilization events for HCV testing. |
